# Meta-analysis of Pregnancy Events in Biomedical HIV Prevention Trials in Sub-Saharan Africa: Implications for Gender Transformative Trials

**DOI:** 10.1007/s10461-024-04459-z

**Published:** 2024-08-17

**Authors:** Lara Lorenzetti, Nhi Dinh, Cason Whitcomb, Andres Martinez, Manju Chatani, Breanne Lievense, Definate Nhamo, Catherine Slack, Natalie Eley, Kathleen MacQueen

**Affiliations:** 1 Behavioral, Epidemiological and Clinical Sciences Division, Durham, NC FHI 360 USA; 2 AVAC, New York, NY USA; 3 Pangaea Zimbabwe, Harare, Zimbabwe; 4 School of Law, University of KwaZulu-Natal, Pietermaritzburg, South Africa

**Keywords:** Meta-analysis, Biomedical HIV prevention trials, Pregnancy, Eligibility criteria, Gender transformative

## Abstract

Historically, pregnant and lactating populations (PLP) have been excluded or disenrolled from biomedical HIV prevention trials, despite being more likely to acquire HIV during pregnancy and the post-partum period. We conducted a meta-analysis of pregnancy events in biomedical HIV prevention trials in sub-Saharan Africa to support trialists moving toward more inclusive clinical and implementation studies. We searched peer-reviewed literature reporting pregnancy events and contraceptive requirements in HIV prevention trials between 2001 and 2022. We hypothesized four variables to explain variation: contraceptive requirements, study start year, study product, and sub-region. We fit a meta-analytic model to estimate individual effect sizes and sampling variances, then conducted sub-group analyses to assess moderating effects. We identified 38 references for inclusion, across which the proportion of pregnancy events was 8% (95% confidence interval [CI]: 6–10%) with high heterogeneity (I^2^ = 99%). Studies not requiring contraceptives (21%, 95%CI: 7–48%) reported a significantly higher proportion of pregnancy events than studies requiring two methods (5%, 95%CI: 2–10%). Studies launched between 2001 and 2007 (11%, 95%CI: 8–16%), microbicide gel trials (12%, 95%CI: 8–18%), and studies conducted in Western Africa (28%, 95%CI: 13–51%) reported higher proportions of pregnancy events than reference groups. Together, these variables have a moderating effect on pregnancy events (p < 0.0001), explaining 63% of heterogeneity in trials. Results describe how, over time, more stringent contraceptive requirements reduced pregnancy events, which ensured necessary statistical power but limited reproductive choice by participants. With the move toward continuing PLP on experimental products, trialists can utilize estimated pregnancy events reported here to inform strategies that accommodate participants’ changing fertility preferences.

## Introduction

Increasingly over the past several decades, HIV prevention programs and studies have adopted rights-based approaches to amplify the voices of and achieve equitable access for marginalized groups [1–4]. Yet, there remains a call for programs and trials to adopt gender transformative approaches, or practices that strengthen systems promoting gender equality [5–7]. At the intersection of these approaches are pregnant and lactating populations (PLP), who have often been categorically excluded from clinical trials. Pregnancy has typically been an exclusion criterion for trial participation, as historically the priority has been to protect the developing fetus from the unknown effects of experimental products [8, 9]. Despite fetal safety concerns for using pregnancy as an exclusion criterion, the result has been a pervasive evidence gap on appropriate prevention and treatment options during pregnancy and breastfeeding [10–14]. This protectionist approach has left pregnant people with uncertain evidence-based prevention options despite being 2–3 times more likely to acquire HIV during pregnancy [15] and 4 times more likely to acquire HIV in the post-partum period [16]. Moreover, a lack of pregnancy-specific pharmacokinetic data has left providers with limited information to ensure PLP and their fetuses are not exposed to inadequate or toxic doses of prevention therapies [17].

Historically, HIV biomedical trials were not prescriptive in terms of contraceptives required. Until recently, in most biomedical HIV prevention trials, a participant who became pregnant was immediately discontinued from the study product and, in many cases, involuntarily disenrolled from the trial [8, 9, 11, 18–20]. As such, pregnancy was interpreted as a liability from a trial implementation standpoint when attrition of study participants due to pregnancy reduced the trial’s power to detect statistically significant differences in effectiveness between products and comparators [9]. A 2013 meta-analysis found that pregnancy rates in microbicide trials were problematically high, leading the authors to recommend the introduction of methods to reduce pregnancy risk to preserve the statistical power of these trials [21]. Around the same time, many biomedical HIV prevention trials began requiring female participants of reproductive age to use highly effective contraceptives (e.g. hormonal oral contraceptive pills, patch, implant, IUD, etc.) [13, 18, 22], which many trials directly provided.

Considering that most biomedical trials require product use for 12 months or longer, it is important to recognize the implication of these requirements on participants’ reproductive choice. Required contraceptive methods may not align with participants’ fertility preferences, which may shift over time [23, 24]. Mandates to be on certain methods for extended periods challenge the rights-based and gender transformative approaches the research community is working toward [13]. Moreover, with the growing need to generate evidence for HIV prevention products for PLP, there is a call to revisit how trialists can modify pregnancy and contraceptive requirements to promote reproductive choice as well as trials’ ultimate aim of testing product effectiveness, thereby reducing the evidence gap [12, 13, 24]. As of mid-2023, two trials were designed and implemented to assess the safety of the dapivirine vaginal ring and oral pre-exposure prophylaxis (PrEP) in pregnancy and during breastfeeding [25]. Additionally, a study evaluating the safety and efficacy of a twice-yearly injectable lenacapavir for PrEP does not require contraception and is allowing participants who become pregnant to remain on the study drug.

To further the conversation about inclusion of PLP in trials, we conducted a meta-analysis of pregnancy data in biomedical HIV prevention trials in sub-Saharan Africa (SSA). We sought to examine pregnancy events by biomedical product, while also considering the effects of contraceptive requirements, region, and time period in which the trial was conducted. Understanding the frequency at which participants become pregnant, including trends in pregnancy rates as contraceptive requirements for participants have evolved, will enable trialists to develop a deeper understanding of how contraceptive requirements impact trial participation and selection bias into trials. Our goal was to support trialists in moving toward gender transformative trials that acknowledge and address participants’ shifting fertility preferences during implementation, while also providing estimates to inform sampling strategies and the inclusion of PLP in trials.

## Methods

We conducted this meta-analysis of reported pregnancy events in biomedical HIV prevention trials in accordance with the Preferred Reporting Items for Systematic Reviews and Meta-analyses (PRISMA) guidelines [26]. A protocol was prospectively registered in PROSPERO (ID# CRD42022334034).

### Databases and Search Terms

With support from a reference librarian, we searched for peer-reviewed literature from five databases representing research on biomedical HIV prevention products from SSA: African Index Medicus, Embase, Global Health, PubMed, and Scopus. Within each database, we searched for literature using the following terms, which include three main constructs: (biomedical prevention products) AND (pregnancy events) AND (sub-Saharan Africa). Appendix A contains a comprehensive list of search terms.

### Eligibility Criteria

To be included in this meta-analysis, an article met all criteria in Table 1. Generally, we included peer-reviewed literature reporting counts or rates of pregnancies among participants in biomedical HIV prevention trials in SSA. Studies were published between 2001 and 2022, representing a period of substantial diversity in products under study. We included studies examining microbicide gels, diaphragms, vaccines, vaginal rings, injectable PrEP, and oral PrEP. We excluded articles reporting on trials of HIV prevention interventions that were not biomedical products, studies conducted exclusively among persons who could not become pregnant, and studies that did not adequately disaggregate key outcomes data by SSA country/site.

**Table 1:** Inclusion and exclusion criteria used for meta-analysis on pregnancy rates in biomedical HIV prevention trials in sub-Saharan Africa

#	Inclusion Criteria	Exclusion Criteria
1	Peer-reviewed articles reporting original, empirical research	Grey literature, reports, abstracts, reviews, blog posts, abstracts
2	English language texts	Texts in languages other than English
3	Articles published between January 2001 and May 2022	Articles published prior to 2001, or after May 2022
4	Articles on biomedical HIV prevention trials with female participants (or persons who can become pregnant), with products including: pre-exposure prophylaxis (oral, injectable, and other forms); microbicide gels, films, foams, and sponges; vaginal rings; HIV vaccines; multi-purpose technologies for preventing HIV and pregnancy	Articles examining prevention interventions that are not biomedical products OR Articles examining biomedical products tested in trials exclusively recruiting men (or persons who cannot become pregnant)
5	Articles reporting a pregnancy measure of interest, even if it is not the main outcome reported by the trial. Outcomes might include: pregnancy counts (overall, or for treatment and control groups), pregnancy incidence, and pregnancy rates. Studies will be included when pregnancy data are presented as related to attrition	Articles that do not include a pregnancy outcome
6	Articles including details on contraceptive requirements (though protocols may be reviewed for this information and/or PIs contacted)	Articles with no information on contraceptive requirements during the clinical trial AND a protocol is not readily available to clarify contraceptive requirements
7	Articles including at least one study site in sub-Saharan Africa	Studies without at least one site in sub-Saharan Africa; if studies in more than one country with only one in sub-Saharan Africa, outcomes data must be disaggregated by country/site

### Citation Screening and Data Management

From each database, we downloaded search results into EndNote files, in which citations were de-duplicated. We then uploaded the de-duplicated list into Covidence, a web-based systematic review data management application. We used a multi-phased screening strategy to determine inclusion. The first stage consisted of a title/abstract review in which articles were flagged as of interest or irrelevant. The second stage consisted of a full text review to ensure all criteria were met. A team of three reviewers ensured that each article was reviewed twice at each stage. Conflicts among reviewers were addressed through discussion until consensus was reached.

Data were extracted independently by two reviewers using a standardized, Excel-based extraction form. Differences in data extraction were resolved through consensus and referral to a third reviewer as necessary. The following information was extracted from each study: (a) study identification: author(s), year of publication; (2) study description: country, trial start and end years, trial name and design, study objectives, biomedical and placebo products examined, contraceptive requirements; (3) outcomes: number of pregnancies events reported overall, and per treatment and control groups, where available, or pregnancy incidence (per 100 person-years). Where necessary, we searched online for study protocols, including via ClinicalTrials.gov, to gather information that was not readily available from the manuscript (e.g., trial contraceptive requirements). Regarding language, since trials referred to participants who can become pregnant as women or female participants (more nuanced gender identity was not reported), data extracted from manuscripts and reported here also describe PLP as women for consistency and to more closely align language with primary results.

### Analysis

Using data extracted from each article, we created an analytic file with pregnancy count information and relevant variables for the meta-analysis. The main outcome for the meta-analysis was pregnancy event count. For articles reporting pregnancy incidence or rates, we re-constructed those figures into pregnancy event data using rate and women-year information from the manuscript, as available. We defined the proportion of pregnancy events as the number of pregnancies over the number of women. This calculation differs from the proportion of women that became pregnant because participants could become pregnant multiple times. Anticipating heterogeneity in our main outcome due to the differences in studies included, we hypothesized four categorical variables may contribute to differences in pregnancy events and were therefore included as control variables in regression models (Table 2): biomedical product, contraceptive requirements, region within SSA, and trial start year.

**Table 2:** Main outcome and control variables included in the meta-analysis

Variable	Response Options
Pregnancy events	Discrete (0—range)
Biomedical HIV prevention product	Categorical: • Microbicide gel (Reference) • Diaphragm/gel • Oral PrEP • Vaginal Ring • Vaccines • Injectable PrEP
Contraceptive requirements	Categorical • not required^a^ (Reference) • Condoms encouraged and/or provided^b^ • An effective method required^c^ • Two methods required^d^
Region within sub-Saharan Africa^e^	Categorical • Southern (Reference) • Eastern • Western • Multi-region^f^
Trial start year	Categorical • 2001–2007 (Reference) • 2008–2014 • 2015–2022

^a^Articles that specifically state that the use of a contraceptive method is not required for trial participation, even if contraceptives are encouraged or provided by the trial

^b^Articles that encourage the use of condoms (or other contraceptives) throughout trial participation, though these may or may not be provided by the trial

^c^Articles that require participants to use at least one effective contraceptive method during trial participation, including hormonal and long-acting methods

^d^Articles that require participants to use two effective contraceptive methods during trial participation, including condoms + one other highly effective method

^e^Regions of sub-Saharan African as defined by the World Health Organization

^f^Trials were defined as multi-region if they contained study sites in more than one sub-region and pregnancy events could not be disaggregated by site (e.g., sites in both southern and eastern regions)

We conducted meta-analyses in R (with metafor [27] and meta packages [28]) using random-effects models to estimate the pooled proportion of pregnancy events over women [29]. First, we calculated the logit transformation of the proportions and their standard errors to achieve a normal distribution [29–31]. We then pooled the individual proportion of pregnancy events and their sampling variances to calculate the weighted average proportion using the inverse variance method. Each study’s weight was calculated as the inverse of that study’s total variance. A study with a larger sample size has less variance and is therefore given more weight, which has a greater impact on the weighted average proportion [29–31]. Finally, we re-converted the logit transformed summary proportions to the original, non-transformed measurement to yield a true summary proportion. Forest plots were created to show the study-specific and pooled point estimate and confidence intervals (CI). Heterogeneity, or the amount of variation in outcomes among included studies, was assessed using the Q test (measuring presence of heterogeneity) [32] and I^2^ statistics (measuring degree of heterogeneity) [33].

We assessed sources of heterogeneity by conducting sub-group analyses and meta-regression by control variables. The sub-group analyses were conducted with a mixed effects model in which the random-effects model was used to combine study effects within each sub-group and the fixed-effect model was used to assess if the effect across the sub-groups varies from each other significantly [29]. For the meta-regression analyses, we first conducted four univariate models separately (e.g., each model tested one of the aforementioned control variables) due to potential multicollinearity resulting from interrelated variables [29, 34]. After finding no multicollinearity, we then ran a multivariate meta-regression model to assess both heterogeneity and the combined moderating effects of the four control variables of interest.

Lastly, we used The Evidence Project risk of bias tool to assess the quality of studies included in this meta-analysis [35]. This tool includes eight criteria: the first three criteria summarize the study design; the next three items focus on sampling and potential biases that may affect equivalence of the study groups or generalizability of the results; and the last two items consider confounding across study arms.

## Results

**Fig. 1:**
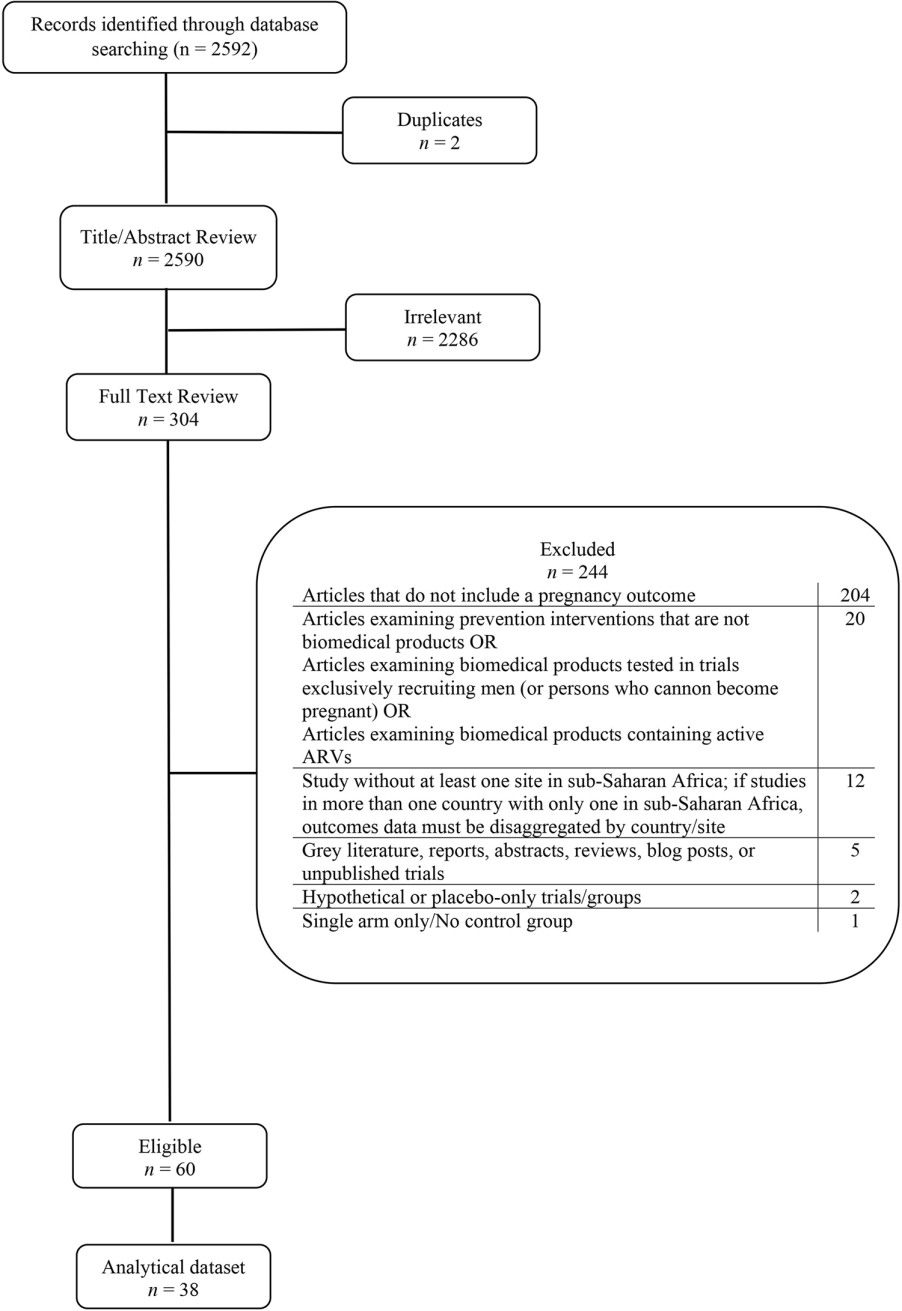
PRISMA Diagram

We identified 2590 unique references through database searches (Fig. 1). A title/abstract review yielded 2286 irrelevant articles, with full texts of the remaining 304 articles reviewed for eligibility. Ultimately, 60 unique studies met all inclusion criteria (Table 3). However, we only included pregnancy data from one publication for each trial, even if multiple publications met the eligibility criteria. This yielded 37 unique references for inclusion, with 38 outcomes by product group in the analytic dataset.

**Table 3:** Variables and pregnancy events by biomedical HIV prevention trial

Trial Name	Associated Articles*	Study Product	Contraceptive Requirements	Trial Start Year	SSA sub-region	# Women	# Pregnancy Events
ACIDFORM Gel Study	Von Mollendorf (2010) [36]	diaphragm/ microbicide gel	Condoms encouraged and/or provided	2001–2007	Southern Africa	120	3
CAPRISA 004	Abdool Karim (2010)* [18] Mathews (2013) [37]	microbicide gel	Effective method required, including hormonal and long-acting methods	2001–2007	Southern Africa	889	54
Carragaurd (Phase II)	Carraguard Study Team (2010) [38]	microbicide gel	Condoms encouraged and/or provided	2001–2007	Southern Africa	400	37
Carraguard (Phase III)	Skoler-Karpoff (2008)* [39] Gaffoor (2016) [40]	microbicide gel	Condoms encouraged and/or provided	2001–2007	Southern Africa	6202	577
Cellulose Sulfate Gel—Zimbabwe (Dia + CS gel/Dia + K) ±	van der Straten (2007) [41]	diaphragm/ microbicide gel	Effective method required, including hormonal and long-acting methods	2001–2007	Eastern Africa	79	6
Cellulose Sulfate Gel—Zimbabwe (CS gel alone/Dia + K) ±s	van der Straten (2007) [41]	microbicide gel	Effective method required, including hormonal and long-acting methods	2001–2007	Eastern Africa	80	11
Cellulose Sulfate Gel—Nigeria	Halpern (2008) [42]	microbicide gel	Condoms encouraged and/or provided	2001–2007	Western Africa	1644	308
DREAM Study (OLE of Ring Study)	Nel (2021) [43]	vaginal ring	Effective method required, including hormonal and long-acting methods	2015–2022	Eastern Africa, Southern Africa	941	26
DS Gel Trial—Uganda	Bakobaki (2005) [44]	microbicide gel	Condoms encouraged and/or provided	2001–2007	Eastern Africa	109	1
FACTS-001	Delany-Moretlwe (2018)* [45] Rees (2021) [46]	microbicide gel	Two methods required, including condoms and other highly effective method	2008–2014	Southern Africa	2059	246
FEM-PrEP	Van Damme (2012)* [47] Todd (2015) [48] Nanda (2016) [49]	daily oral PrEP	Effective method required, including hormonal and long-acting methods	2008–2014	Eastern Africa, Southern Africa	2056	125
HIVIS03	Bakari (2011) [50]	vaccine	Condoms encouraged and/or provided	2001–2007	Eastern Africa	15	1
HPTN 035	Balkus (2016) [51]	microbicide gel	Contraceptives not required	2001–2007	Eastern Africa, Southern Africa	2830	295
HPTN 084	Delany-Moretlwe (2022) [52]	injectable PrEP	Effective method required, including hormonal and long-acting methods	2015–2022	Eastern Africa, Southern Africa	3224	29
HVTN 086/SAAVI 103	Churchyard (2016) [53]	vaccine	Two methods required, including condoms and other highly effective method	2008–2014	Southern Africa	95	1
HVTN 097	Gray (2019) [54]	vaccine	Two methods required, including condoms and other highly effective method	2008–2014	Southern Africa	42	2
HVTN 100	Laher (2020) [55]	vaccine	Two methods required, including condoms and other highly effective method	2015–2022	Southern Africa	91	2
HVTN 503/Phambili Study	Gray (2011)* [56] Latka (2012) [57] Mpofu (2021) [58]	vaccine	Two methods required, including condoms and other highly effective method	2001–2007	Southern Africa	360	66
HVTN 702	Gray (2021) [59]	vaccine	Two methods required, including condoms and other highly effective method	2015–2022	Southern Africa	3786	163
HVTN 703/HPTN 081	Mgodi (2021) [60]	vaccine	Condoms encouraged and/or provided	2015–2022	Eastern Africa, Southern Africa	1924	131
MDP 301	McCormack (2010)* [61] Abaasa (2013) [62] Crook (2014) [63] Moodley (2016) [22]	microbicide gel	Condoms encouraged and/or provided	2001–2007	Eastern, Southern Africa	9385	595
Methods for Improving Reproductive Health in Africa (MIRA)	Padian (2007)* [64] Blanchard (2011a) [65] Blanchard (2011b) [66] McCoy (2013) [67]	diaphragm/ microbicide gel	Two methods required, including condoms and other highly effective method	2001–2007	Southern Africa	5039	108
MTN 003 (VOICE)	Marrazzo (2015)* [20] Akello (2017) [68]	daily oral PrEP	Effective method required, including hormonal and long-acting methods	2008–2014	Eastern Africa, Southern Africa	5029	430
MTN-020/ASPIRE; MTN-016	Makanani (2018)* [69] Balkus (2017) [70]	vaginal ring	Effective method required, including hormonal and long-acting methods	2008–2014	Eastern Africa, Southern Africa	2629	179
Partners PrEP	Mugo (2014)* [71] Baeten (2012) [19] Mugwanya (2013) [72] Matthews (2014) [20] Murnane (2014) [73]	daily oral PrEP	Condoms encouraged and/or provided	2008–2014	Eastern Africa	1785	288
Pilot Study of Pre-Exposure Prophylaxis (PrEP) to Evaluate Safety, Acceptability, and Adherence in At-risk Populations in Uganda	Kibengo (2013) [74]	daily oral PrEP	Effective method required, including hormonal and long-acting methods	2008–2014	Eastern Africa	36	3
rAAV2-HIV-1 Subtype C Vaccine Study	Vardas (2010) [75]	vaccine	Effective method required, including hormonal and long-acting methods	2001–2007	Eastern Africa, Southern Africa	46	6
RV 172	Kibuuka (2009)* [76] Kibuuka (2010) [77]	vaccine	Effective method required, including hormonal and long-acting methods	2001–2007	Eastern Africa	103	9
SAVVY—Ghana	Peterson (2007) [78]	microbicide gel	Condoms encouraged and/or provided	2001–2007	Western Africa	2142	942
SAVVY—Nigeria	Feldblum (2008) [79]	microbicide gel	Condoms encouraged and/or provided	2001–2007	Western Africa	2153	552
TaMoVac 01 (Phase I)	Joachim (2016) [80]	vaccine	Condoms encouraged and/or provided	2008–2014	Eastern Africa	15	1
TAMOVAC 01 (Phase IIa)	Munseri (2015) [81]	vaccine	Condoms encouraged and/or provided	2008–2014	Eastern Africa	54	3
TaMoVac II	Viegas (2018) [82]	vaccine	Effective method required, including hormonal and long-acting methods	2008–2014	Eastern Africa	97	11
TDF2 Study	Thigpen (2012)* [83] Kebaabetswe (2015) [84] Gust (2016) [85]	daily oral PrEP	Effective method required, including hormonal and long-acting methods	2001–2007	Southern Africa	557	107
IPM 015 (Phase I/II)	Nel (2016a) [86]	vaginal ring	Effective method required, including hormonal and long-acting methods	2008–2014	Eastern Africa, Southern Africa	280	6
Ring Study (Phase III)	Nel (2016b) [87]	vaginal ring	Effective method required, including hormonal and long-acting methods	2008–2014	Eastern Africa, Southern Africa	1959	48
V001	Joako (2010) [88]	vaccine	Effective method required, including hormonal and long-acting methods	2001–2007	Eastern Africa	43	3
Vaccine LTFU Study	Schmidt (2014) [89]	vaccine	Contraceptives not required	2001–2007	Eastern Africa, Southern Africa	91	34

*Primary article reporting pregnancy outcomes data ± van der Straten [41] provided pregnancy events disaggregated by product groups, which we were able to analyze separately

### Description of Studies

**Fig. 2:**
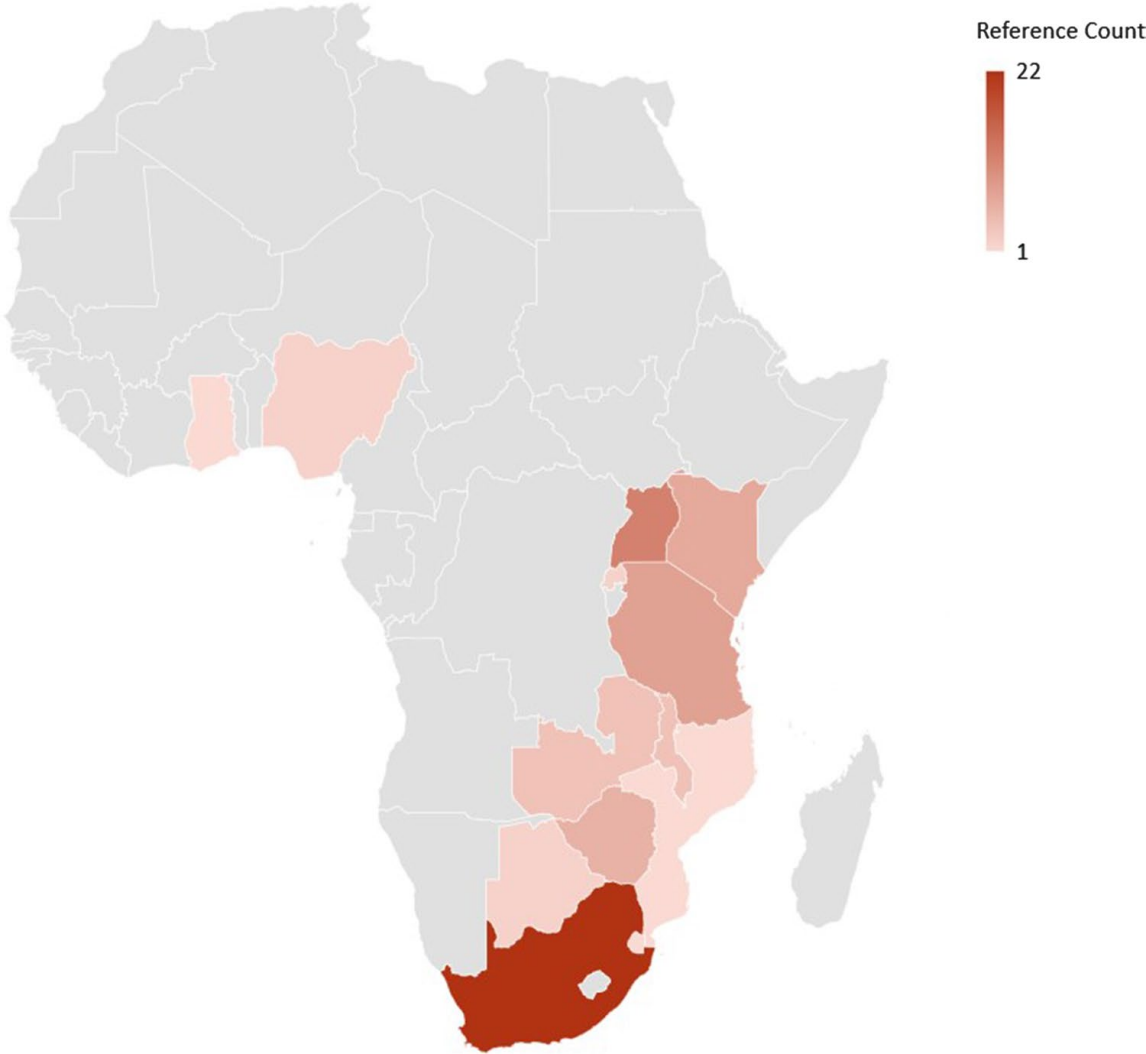
Map depicting study locations included in the meta-analysis

Of references included in the meta-analysis, 37% were from vaccine trials, with the second highest proportion reporting on microbicide trials. About half of studies (53%) began between 2001 and 2007 and only 13% reported a study start year between 2015 and 2022. Approximately one-third of references (31%) describe studies in Southern Africa, while 29% and 8% describe studies in Eastern and Western Africa, respectively. A map outlining country locations for included references is available in Fig. 2. An additional 32% were characterized as “multi-region” or having one site in various sub-regions of SSA. In terms of contraceptive requirements, 42% of studies required an effective method, including hormonal and long-acting methods, with an additional 19% requiring at least two methods. There were 5% of studies that did not require a contraceptive and 34% that encouraged condom use.

### Quality Assessment

Summary scores for all references included in this meta-analysis are presented in Appendix B. All studies fully met the three criteria on study design. The two criteria assessing potential confounding across study arms were scored highly, with only one study not reporting comparisons of sociodemographic measures across groups at baseline [75]. For the sampling criteria, while all studies met the requirement on random selection of participants to the intervention, none met the requirement on random selection of participants for assessments. In terms of follow-up rate, among the 35 studies that reported these results, only five studies did not meet the retention rate of 80% or more [38, 42, 53, 79, 82].

### *Meta*-analysis

**Fig. 3:**
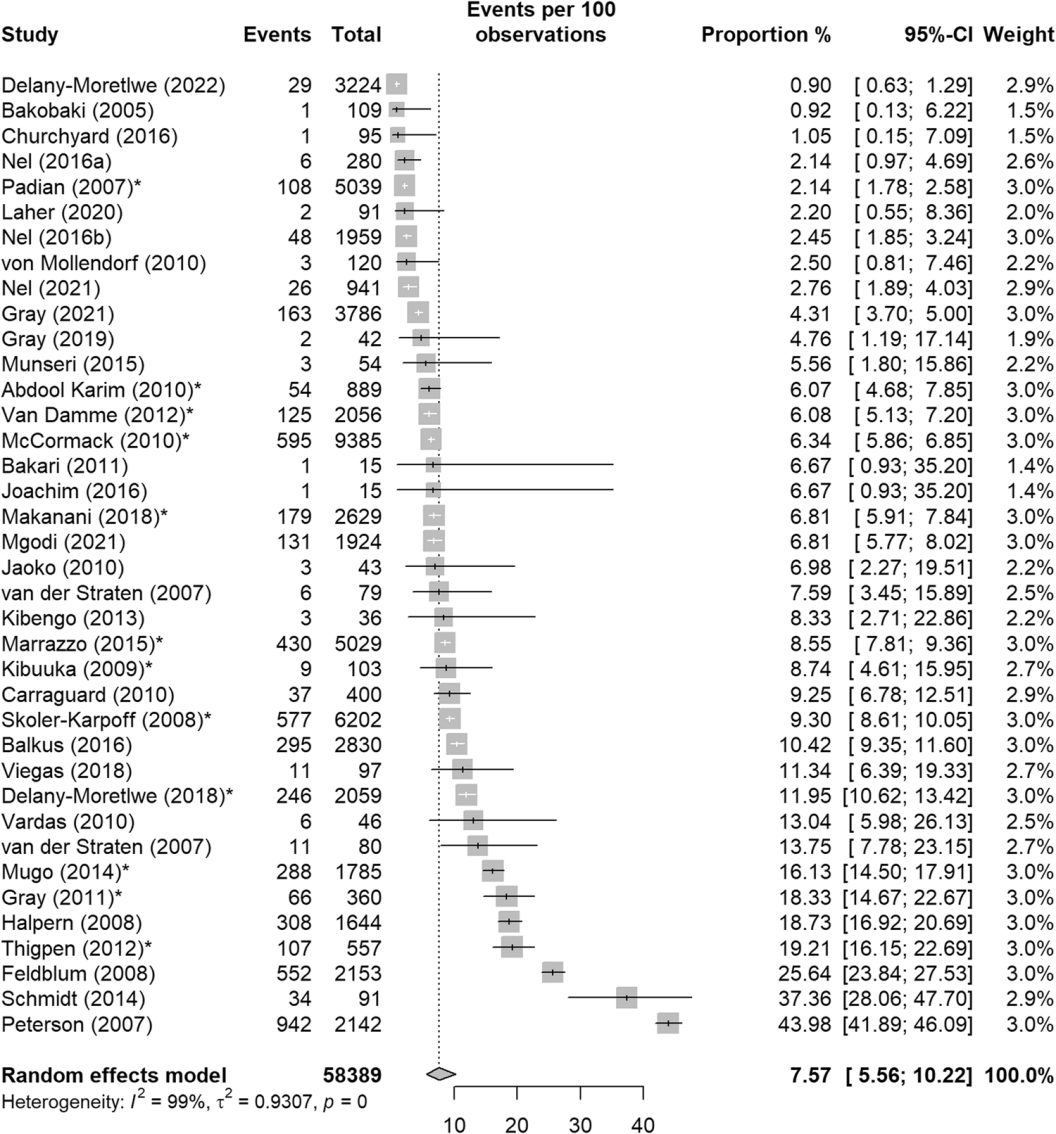
Pooled proportion of pregnancy events from HIV biomedical prevention trials in sub-Saharan Africa, 2001–2022. *CI* confidence interval; *I2* the percentage of total variation across studies that is due to heterogeneity rather than chance; *τ2* tau-squared is an estimate of the between-study variance; *p* p-value

Overall, the estimated pooled proportion of pregnancy events across included studies was 7.57% (95%CI: 5.56–10.22%) with high heterogeneity (Q = 3750.35, p < 0.01 and I^2^ = 99.04%). Figure 3 depicts the forest plot with the pooled estimate of all included studies. Results of the sub-group analysis and meta-regression are shown in Table 4.

**Table 4:** Summary of estimated proportions of pregnancy events by study product, contraceptive requirements, sub-Saharan Africa sub-region, and trial start year, 2001—2022

	Number of Studies	Pooled Proportion of Pregnancy Events (%) (95% CI)	Meta-regression: Univariate analysis (Odd Ratio, 95% CI)
Overall	38	7.57 (5.56, 10.22)	
Study product			
Microbicide gel	11	12.22 (8.04, 18.16)	Ref
Diaphragm/gel	3	3.37 (1.32, 8.36)	0.25 * (0.09, 0.73)
Oral PrEP	5	10.95 (5.80, 19.73)	0.88 (0.38, 2.04)
Vaginal Ring	4	3.25 (1.53, 6.79)	0.24** (0.10, 0.60)
Vaccines	14	8.36 (5.36, 12.81)	0.66 (0.34, 1.28)
Injectable PrEP	1	0.90 (0.63, 1.29)	0.07*** (0.01, 0.32)
Contraceptive Requirements			
Contraceptives not required	2	20.51 (6.71, 48.08)	Ref
Condoms encouraged and/or provided	13	10.25 (6.21, 16.46)	0.44 (0.11, 1.78)
Effective method required	16	6.18 (3.95, 9.53)	0.26 (0.07, 1.00)
Two methods required	7	5.05 (2.45, 10.13)	0.21* (0.05, 0.91)
SSA sub-region			
Southern	12	6.40 (3.91, 10.32)	Ref
Eastern	11	8.28 (4.72, 14.11)	1.32 (0.60, 2.92)
Western	3	28.41 (13.24, 50.78)	5.80** (1.96, 17.22)
Multi-region	12	5.85 (3.67, 9.21)	0.91 (0.44, 1.86)
Trial start year			
2001–2007	20	10.98 (7.66, 15.51)	Ref
2008–2014	13	6.31 (3.88–10.12)	0.55 (0.29, 1.05)
2015–2022	5	2.86 (1.33, 6.05)	0.24** (0.10, 0.57)

*Statistically significant at p < 0.05, ** statistically significant at p < 0.01, and *** statistically significant at p < 0.001 with differences compared to the reference group only

### Study Product

**Fig. 4:**
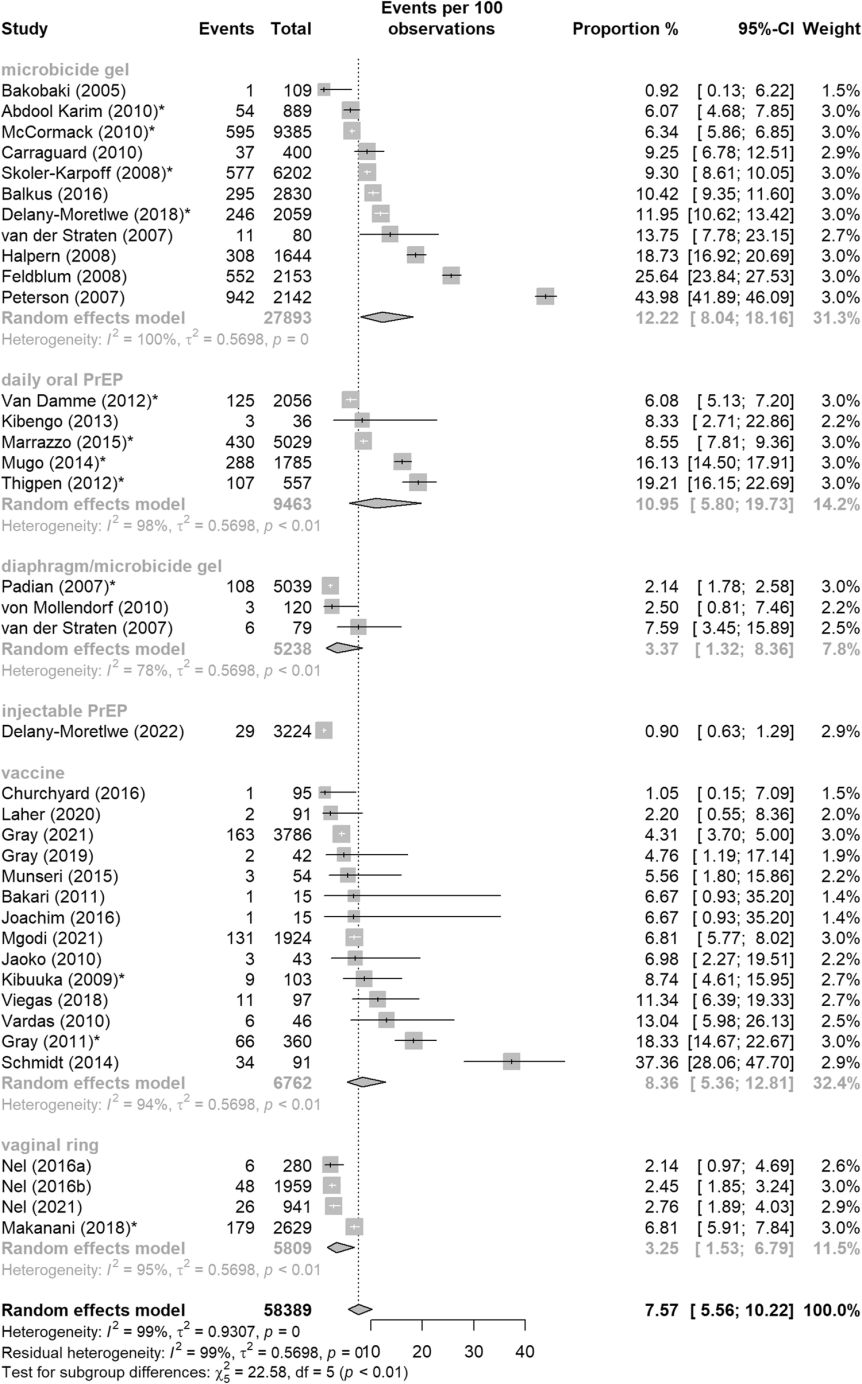
Pooled proportion of pregnancy events from HIV biomedical prevention trials in sub-Saharan Africa, 2001–2022 by trial’s product type: (1) Microbicide gel, (2) Daily oral PrEP, (3) Diaphragm/microbicide gel, (4) Injectable PrEP, (5) Vaccine, and (6) Vaginal ring

When examining pregnancy events by study product, the highest proportion of pregnancy events was among microbicide gel studies (12.22%, 95%CI: 8.04–18.16%) and oral PrEP trials (10.95%, 95%CI: 5.80–19.73%), with the lowest rate within a single injectable PrEP study (0.90%, 95%CI: 0.63–1.29%) (Fig. 4). Compared to microbicide gel studies, the odds of a pregnancy event in diaphragm/microbicide gel studies was 75% lower (OR = 0.25, 95%CI: 0.09–0.73, p = 0.01), 93% lower in the injectable PrEP study (OR = 0.07, 95%CI: 0.01–0.32, p < 0.001), and 76% lower in vaginal ring studies (OR = 0.24, 95%CI: 0.10–0.60, p < 0.01) (Table 4). These differences were statistically significant. Although the odds of a pregnancy event in oral PrEP studies and in vaccine studies were lower than microbicide gel studies, these differences were not statistically significant.

### Contraceptive Requirements

**Fig. 5:**
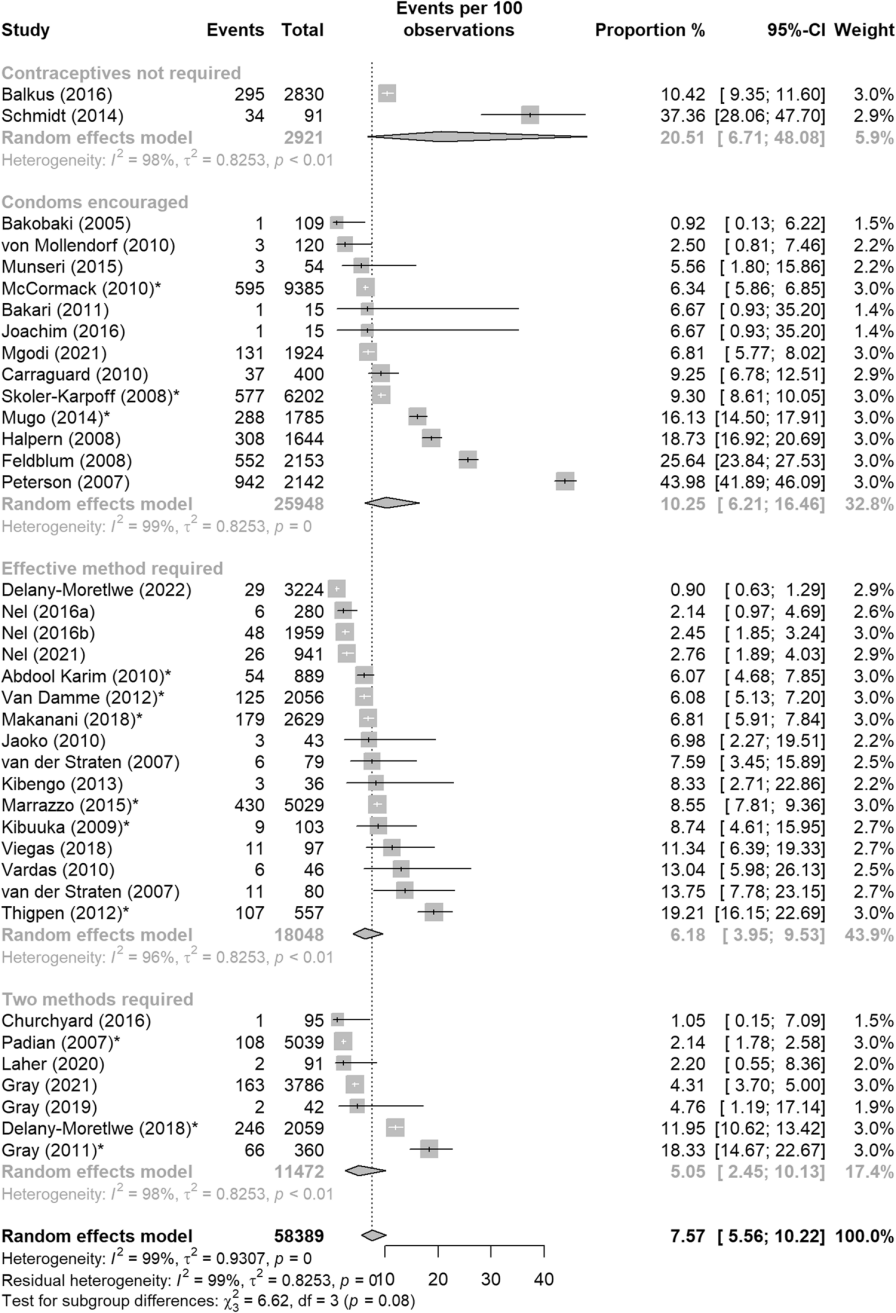
Pooled proportion of pregnancy events from HIV biomedical prevention trials in sub-Saharan Africa, 2001–2022 by trial’s contraceptive requirement: (1) Contraceptive not required, (2) Condoms encouraged, (3) Effective method required, and (4) Two effective methods required

The pooled proportions of pregnancy events decreased with stricter contraceptive requirements. This proportion was highest among studies without contraceptive requirements (20.51%, 95%CI: 6.71–48.08%) (Fig. 5), followed by condoms encouraged and/or provided (10.25%, 95%CI: 6.21–16.46%) and one effective method required (6.18%, 95%CI: 3.95–9.53%), and was lowest among studies that required two contraceptive methods (5.05%, 95%CI: 2.45–10.13%). Compared to the contraceptives not required sub-group, the odds of a pregnancy event in the two methods required group was 79% lower, which was significant (OR = 0.21, 95%CI: 0.05–0.91, p = 0.04) (Table 4). The odds of a pregnancy event in the condoms encouraged or one effective methods sub-groups was not significantly different from no contraceptives required.

### SSA Sub-region

**Fig. 6:**
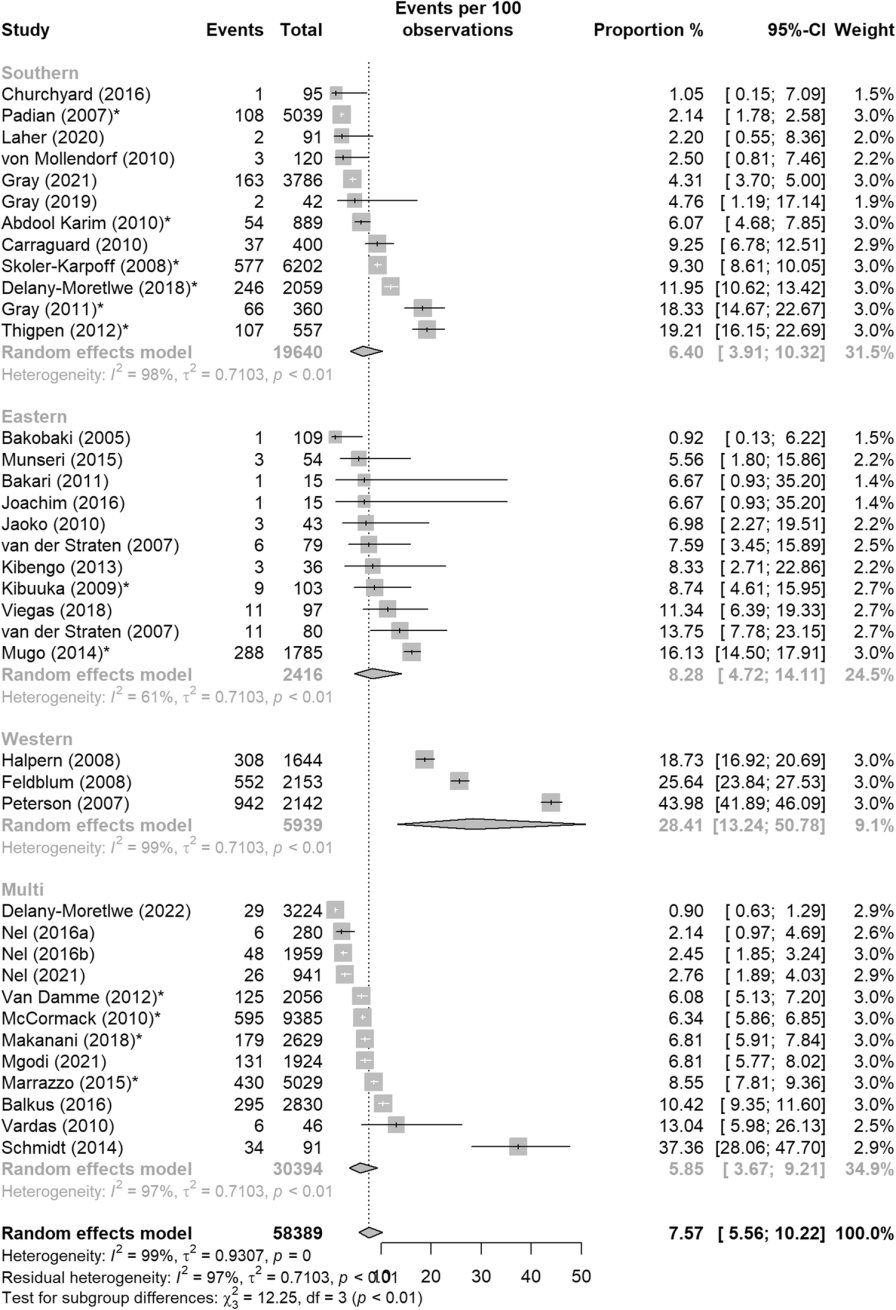
Pooled proportion of pregnancy events from HIV biomedical prevention trials in sub-Saharan Africa, 2001–2022 by sub-region: (1) Southern, (2) Eastern, (3) Western, and (4) multi-region

The pooled proportion of pregnancy events across studies in Southern Africa was 6.40% (95%CI: 3.91–10.32%), 8.28% in Eastern Africa (95%CI: 4.72–14.11%), 28.41% in Western Africa (95%CI: 13.24–50.78%), and 5.85% in multi-regional references (95%CI: 3.67–9.21%) (Fig. 6). The odds of a pregnancy event in the Western sub-group were about 6 times the odds in the Southern sub-group, which was significant (OR = 5.80, 95%CI: 1.96–17.22, p < 0.01) (Table 4). There was no significant difference in the odds of a pregnancy event between studies in the Eastern or multi-region sub-groups compared to the Southern sub-group.

### Trial Start Year

**Fig. 7:**
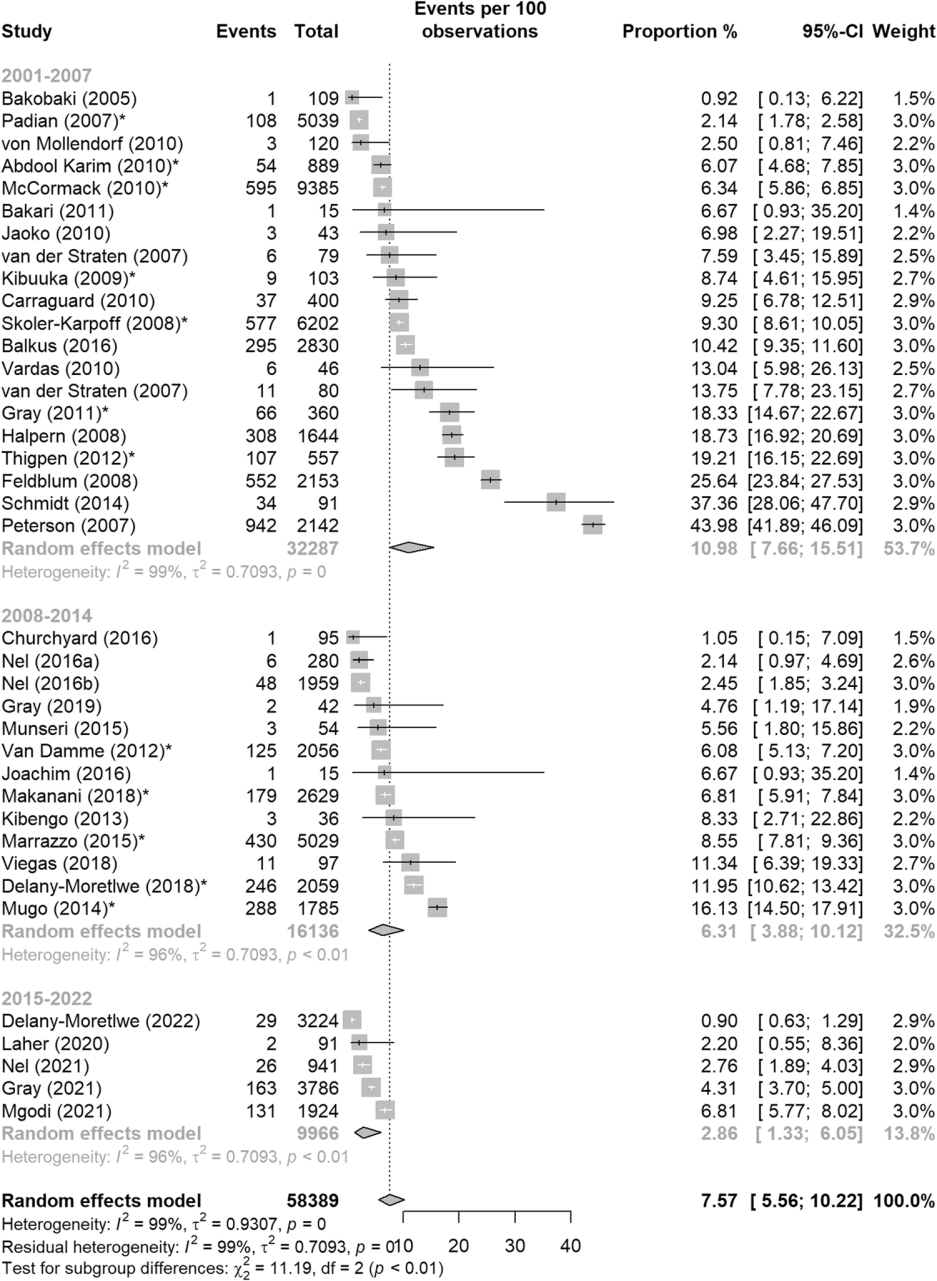
Pooled proportion of pregnancy events from HIV biomedical prevention trials in sub-Saharan Africa, 2001–2022 by trial’s starting year: (1) 2001–2007, (2) 2008–2014, and (3) 2015–2022

The pooled proportion of pregnancy events decreased over time from an estimated 10.98% (95%CI: 7.66–15.51%) across studies started within 2001–2007, to 6.31% (95%CI: 3.88–10.12%) from 2008 to 2014, and 2.86% from 2015 to 2022 (95%CI: 1.33–6.05%) (Fig. 7). The odds of a pregnancy event across studies that started between 2015 and 2022 was 76% lower than studies started between 2001 and 2007, which was significantly different (OR = 0.24, 95%CI: 0.10–0.57, p < 0.01) (Table 4). There was no significant difference in the odds of a pregnancy event between studies starting within 2001–2007 and 2008–2014.

### *Meta*-regression Results

Multiple meta-regression results indicate that product type, contraceptive requirement, SSA sub-region, and trial’s starting year together account for 63.17% of heterogeneity and have a significant moderating effect (QM(13) = 60.91, p < 0.0001). However, there was still a significant amount of residual heterogeneity that was not explained by these moderators (QE(24) = 533.57, p < 0.0001).

## Discussion

Challenging fetal safety considerations, the historic exclusion of PLP from biomedical HIV prevention trials created an evidence gap around effective prevention options during pregnancy and breastfeeding [10–14]. In addition to the increased risk of HIV acquisition for PLP during these periods, acute maternal infection is associated with elevated risk of perinatal transmission [90]. Furthermore, in the absence of pregnancy-specific pharmacokinetic data, PLP and the developing fetus are at risk of receiving inadequate or even toxic doses of prevention medication, resulting in further harm [17]. Recent guidance has recommended changes within the ethical framing of research among PLP, including shifting from protecting PLP *from* research to protecting them *through* research [91]. Leading ethics guidelines strongly recommends that trialists strive toward a more equitable evidence base for under-represented groups [92] and review and address critical pregnancy-related evidence gaps [93].

We conducted this meta-analysis with the goal of providing insight into addressing participants’ shifting fertility preferences over the course of a study’s implementation period while also ensuring that studies remain adequately powered despite pregnancy-related attrition or inclusion of PLP. Over more than two decades covering 38 biomedical HIV prevention trials, we found an estimated pooled proportion of pregnancy events at nearly 8%, with heterogeneity partially explained by differences in study product, contraceptive requirements, region within SSA, and trial start year. Within each of these variables, the highest proportions of pregnancy events were attributed to microbicide trials, trials without contraceptive requirements, trials in Western Africa, and those launched between 2001 and 2007.

These results capture a complicated history surrounding pregnancy and HIV prevention trials. Notably, results indicate that earlier trials, especially microbicide gel trials in Western Africa, had higher proportions of pregnancy events than more recent trials. This can likely be attributed, in part, to more restrictive contraceptive requirements that have been applied by trials over time. That microbicide gel trials have higher proportions of pregnancy events than other products is likely confounded by both time and contraceptive requirements. A lack of access to contraceptives and high rates of pregnancy likely undermined the SAVVY and Cellulose Sulfate trials conducted in Western Africa, which were stopped early due to lack of effectiveness [79, 94]. Around this time, there was fervent discussion about the need for greater contraceptive requirements in trials [9]. An earlier meta-analysis that estimated high pregnancy rates in microbicide trials through 2012 (23.4 pregnancies per 100 woman-years) [21], also called for more comprehensive contraceptive requirements to improve sample size and power. As results of our analysis demonstrate, over time, trials typically moved toward requiring the use of a highly effective, non-barrier method, and in some cases two methods (one highly effective method plus condoms). However, even when two contraceptive methods were required, pregnancies still occurred in trials.

Regarding geographic disparities, we found that of three trials in Western Africa, where pregnancy events were significantly higher than other regions, all examined microbicide gels between 2001 and 2007. Thereafter, no trials were conducted in the region, which raises questions about regional representation within biomedical trials. Part of the motivation for conducting more recent trials in Eastern and Southern Africa may be due to academic affiliations or a need to conduct research in areas with growing HIV epidemics. The change in HIV prevalence in Southern Africa was much greater than in Western Africa over the past 20 years [95]. However, trials may also have shifted to Eastern and Southern Africa where contraceptive prevalence rates have historically been higher [96, 97], given the increased emphasis on contraceptive requirements over time.

Considering the increasing trend toward inclusion of PLP in trials, there is much to be learned from this history regarding the nuanced context against which people of reproductive potential participate in trials. Trialists must consider the multiple motivations behind individuals’ willingness to participate in prevention trials, as reasons for participation are multi-faceted and are not limited to addressing HIV prevention needs. Preventing acquisition of HIV and altruistic desires to support the community are commonly noted as primary motivators for participation [98], and yet qualitative research elucidates a more complex story. Individuals are also motivated by the potential for personal health benefits offered through trials, such as free health care and consistent HIV testing [99–101]. The VOICE trial of a vaginal gel with very low adherence but high retention found that participants quantitatively reported altruism as a primary motivator for participation, but qualitatively stated they were driven by the potential personal health benefits offered by the trial [100]. Qualitative interviews following the FEM-PrEP trial, another trial with low adherence and high retention, found that participants valued the access to monthly HIV testing even more than the access to the daily prevention product [102]. Cultural context and gendered social dynamics also directly influence participants’ desire to participate in trials or adhere to trial requirements [103], including contraceptive requirements. Some women may have constraints on decision-making autonomy regarding fertility preferences, and in SSA where fertility rates are generally high [104], they may feel societal or familial pressure to become pregnant [105, 106].

Trial teams should balance participants’ myriad reasons for trial participation, as well as the need for evidence on safe and effective HIV prevention options for PLP. It is recommended that trial teams embrace a more gender transformative approach [107] to trials, which generally argues for adopting strategies that critically examine gender norms and dynamics while also strengthening systems that support gender equality. Applied to HIV prevention trials, trialists should responsibly loosen contraceptive requirements and allow for greater reproductive choice during trials. Where appropriate, this may include dropping the contraceptive requirement altogether or recruiting individuals who are actively seeking to become pregnant. A gender transformative approach would moreover ensure a comprehensive contraceptive method mix through direct provision rather than referrals. Many protocols reviewed for this meta-analysis stated that contraceptives were provided “per local standards,” which indicates inconsistent strategies for participant access to contraceptives across trials and sites within trials [108]. Instead, trialists should outline in protocols exactly which contraceptive methods will be available to participants via which strategy, and how participants will be guided to select their choice of contraceptive at the same visit during which they receive the study product.

Participants should also be permitted and supported to switch to contraceptive methods that better suit their needs and lifestyle over the course of the study. Apart from aligning with gender transformation, it may also have a direct impact on participant retention and adherence in prevention trials. Participation in the MTN-020/ASPIRE phase III trial required the use of highly effective contraceptive methods [69, 70]. ASPIRE improved uptake of long-acting reversible contraceptive methods with counseling and immediate access to at least four different contraceptive options, either delivered directly at the site or referred out to the public sector [109]. A qualitative component of APSIRE further recommended that trialists pre-emptively address concerns around potential impacts of contraceptive and study product on fertility to further support adherence to study product [110].

As trial teams responsibly loosen restrictions on contraceptive requirements, and therefore better prepare for pregnancies within trials, we also recommend that trial teams clearly outline in protocols the steps to be taken once a pregnancy is identified. Since consent forms were not reviewed as a part of this analysis, it is unclear the extent to which this information is consistently and clearly communicated to participants before a pregnancy occurs. Standard practice has been to remove participants from study products and, in some cases, disenroll them completely from the trial. For studies in which pregnancy would require discontinuation of study products, trialists should consider re-enrolling participants once they enter their postpartum period, if they do not already do so.

However, to better inform prevention options for PLP, certain trials should be encouraged to actively enroll PLP and re-consent those who become pregnant and who may be willing to stay on experimental products. Doing so would support the gathering of pre- or early-clinical data from PLP, contributing valuable information prior to drug authorization. However, inclusion of PLP may not be appropriate for all trials or trial phases. Leading international ethics guidelines recommend that research in PLP should only be initiated after careful consideration of the “best available data” from pre-clinical research in pregnant animal models, research in non-pregnant women, retrospective observational studies, and pregnancy registries, and when a favorable risk–benefit ratio is met [111]. For those trials in which PLP will be enrolled and encouraged to stay on product, protocols should clearly outline any additional monitoring or support participants would receive, including referrals or direct provision of antenatal care. The WHO/IMPAACT/IAS Call to Action and the AVAC/PHASES Think Tank report endorsed that product developers should remove contraception requirements in pre-licensure trials and allow participants who become pregnant to re-consent to stay on study drug and contribute pregnancy pharmacokinetic and safety data, once non-clinical reproductive toxicity data indicate no concerning signals and dosing for nonpregnant people is determined [112, 113]. In addition, the AVAC/PHASES Think Tank report recommended that HIV prevention trial sites actively facilitate and strongly consider providing access to sexual and reproductive health services that promote women’s health and autonomy, including contraceptive counseling, even when contraception requirements are not warranted [112]. A further consideration for trials including PLP is the need to track and examine safety data for PLP and their fetuses, which would inevitably include data on abortion. Collecting such data may be politically or legally onerous to collect in the many contexts where biomedical HIV prevention research is conducted given the growing restrictions on access to safe and legal abortions [114].

We aimed to highlight the need for more gender transformative approaches for PLP, and to support trial teams as they can use the results of this meta-analysis to better prepare for pregnancy in trials. There are benefits to moving toward gender transformative trials for trial teams, not just participants. The responsible loosening of contraceptive requirements and better preparing for pregnancies in trials will allow trial teams to generate much needed evidence on prevention strategies for PLP and the developing fetus. Moreover, current restrictions on pregnancy likely lead to selection bias into trials, namely, the inclusion of participants who do not plan to become pregnant. By making trials more inclusive, trial teams can attract a wider mix of participants that are more representative of the average end-user. Trial teams can use these results to estimate attrition rates from trials due to pregnancy given certain requirements, which can be considered in power calculations and sampling plans.

### Limitations

This meta-analysis provides a comprehensive overview of pregnancy events in biomedical HIV prevention trials over the past two decades. We used a rigorous approach to identify articles for inclusion in this meta-analysis; notwithstanding, certain articles or trials may have been inadvertently excluded. A limitation of this meta-analysis is that the sub-group analyses are essentially bivariate analyses and do not take confounding into consideration. As such, interpreting the estimated proportion of pregnancy by year, for example, may be confounded by the fact that contraceptive requirements changed over time. However, the final multivariable meta-regression helps to address confounding of key variables. Also, although we anticipated heterogeneity in this meta-analysis—and sought to identify and prioritize certain variables that may contribute to this variation in pregnancy events—the four variables identified did not account for all heterogeneity. There may be other important considerations, and we encourage future meta-analyses to examine average participant age and trial phase as additional key variables contributing to variation in pregnancy events. Future studies might also consider how country-specific abortion laws contribute to heterogeneity, as access to safe and legal abortion may influence pregnancy events and other outcomes or safety data for PLP and their fetuses. Lastly, the validity of these results is dependent on the data quality from the original studies, though our assessment indicates generally low risk of bias for included studies. Further, we acknowledge that there could be publication bias inherent to the papers included. However, publication bias is more challenging to identify and quantify when the focus of the meta-analysis is not the main outcome of abstracted articles [115]. This meta-analysis reviewed a non-primary outcome (e.g., pregnancy events), making tests to assess publication bias less interpretable. Notably, as publication bias is typically an issue of small study effects [116], we highlight that 84% of studies included in this review had sample sizes greater than 50.

## Conclusions

This meta-analysis found a roughly 8% estimated proportion of pregnancy events in biomedical HIV prevention trials in SSA over the past 20 years. Study product, contraceptive requirements, region where the study took place, and trial start year were all important in contributing to heterogeneity in the proportion of observed pregnancy events. Trial teams are faced with various responsibilities, including generating a scientific evidence base for this at-risk population as well as accommodating participant fertility choices. Trial teams can use these findings to reflect on the reality and motivations with which participants join trials, to adopt strategies that support participants’ shifting fertility preferences during trials, and to improve planning for pregnancies during trials, including implications for sampling, improving study power, and overall design. This might include better preparation for attrition due to pregnancy or for the opportunity for PLP to remain on study product, where ethics recommendations are met. Taken together, these considerations would provide a more gender transformative approach to how pregnancy is managed in biomedical HIV prevention trials.

## Data Availability

The data supporting the findings of this study are available within the article [and/or] its supplementary materials.

## Appendix A

See Table 5.

**Table 5:** Search terms used for meta-analysis on pregnancy rates in biomedical HIV prevention trials in sub-Saharan Africa

Biomedical HIV prevention products	(“HIV prevention” OR “preventing HIV” OR anti-retroviral agents OR pre-exposure prophylaxis OR “PrEP” OR “injectable PrEP” OR “long-acting injectable PrEP” OR ((“prevention and control” OR “microbicide gel” OR “microbicide sponge” OR “microbicide film” OR “microbicide foam” OR “vaginal ring” OR “dapivirine ring” OR multipurpose technolog* OR “MPTs”) AND HIV))
Clinical trials	(clinical trial OR “biomedical HIV prevention trial” OR “biomedical HIV prevention trials” OR “HIV prevention trial”)
Pregnancy rates	pregnant OR “pregnancy rate” OR “pregnancy rates” OR “pregnancy incidence”
Sub-Saharan Africa	Sub-Saharan Africa OR Angola OR Benin OR Botswana OR “Burkina Faso” OR Burundi OR Cameroon OR “Central African Republic” OR Chad OR Congo OR “Côte d'Ivoire” OR OR “Democratic Republic of Congo” OR Djibouti OR “Equatorial Guinea” OR Eritrea OR Eswatini OR Ethiopia OR Gabon OR Gambia OR Ghana OR Guinea OR “Guinea Bisau” OR Kenya OR Lesotho OR Liberia OR Madagascar OR Malawi OR Mali OR Mauritania OR Mauritius OR Mozambique OR Namibia OR Niger OR Nigeria OR Rwanda OR Ruanda OR Senegal OR “Sierra Leone” OR Somalia OR “South Africa” OR “South Sudan” OR Sudan OR Swaziland OR Tanzania OR Togo OR Uganda OR Zambia OR Zimbabwe

## Appendix B

See Table 6.

**Table 6:** Completed Evidence Project risk of bias tool on studies included in the meta-analysis on pregnancy rates in biomedical HIV prevention trials in sub-Saharan Africa

Author Year	1. Cohort	2 Control or comparison group	3 Pre/post intervention data	4 Random assignment of participants to the intervention	5 Random selection of participants for assessment	6 Follow-up rate of 80% or more	7 Comparison groups equivalent on sociodemographic	8 Comparison groups equivalent at baseline on outcome measures
von Mollendorf (2010) [36]	Yes	Yes	Yes	Yes	No	Yes	Yes	Yes
Abdool Karim (2010)* [18]	Yes	Yes	Yes	Yes	No	Yes	Yes	Yes
Carraguard (2010) [38]	Yes	Yes	Yes	Yes	No	No	Yes	Yes
Skoler-Karpoff (2008)* [39]	Yes	Yes	Yes	Yes	No	Yes	Yes	Yes
van der Straten (2007) [41]	Yes	Yes	Yes	Yes	No	Yes	Yes	Yes
van der Straten (2007) [41]	Yes	Yes	Yes	Yes	No	Yes	Yes	Yes
Halpern (2008) [42]	Yes	Yes	Yes	Yes	No	No	Yes	Yes
Nel (2021) [43]	Yes	Yes	Yes	Yes	No	Yes	Yes	Yes
Bakobaki (2005) [44]	Yes	Yes	Yes	Yes	No	Yes	Yes	Yes
Delany-Moretlwe (2018)* [45]	Yes	Yes	Yes	Yes	No	Yes	Yes	Yes
Van Damme (2012)* [47]	Yes	Yes	Yes	Yes	No	Yes	Yes	Yes
Bakari (2011) [50]	Yes	Yes	Yes	Yes	No	Not Reported	Yes	Yes
Balkus (2016) [51]	Yes	Yes	Yes	Yes	No	Yes	Yes	Yes
Delany-Moretlwe (2022) [52]	Yes	Yes	Yes	Yes	No	Yes	Yes	Yes
Churchyard (2016) [53]	Yes	Yes	Yes	Yes	No	No	Yes	Yes
Gray (2019) [54]	Yes	Yes	Yes	Yes	No	Yes	Yes	Yes
Laher (2020) [55]	Yes	Yes	Yes	Yes	No	Yes	Yes	Yes
Gray (2011)* [56]	Yes	Yes	Yes	Yes	No	Yes	Yes	Yes
Gray (2021) [59]	Yes	Yes	Yes	Yes	No	Yes	Yes	Yes
Mgodi (2021) [60]	Yes	Yes	Yes	Yes	No	Yes	Yes	Yes
McCormack (2010)* [61]	Yes	Yes	Yes	Yes	No	Yes	Yes	Yes
Padian (2007)* [64]	Yes	Yes	Yes	Yes	No	Yes	Yes	Yes
Marrazzo (2015)* [20]	Yes	Yes	Yes	Yes	No	Yes	Yes	Yes
Makanani (2018)* [69]	Yes	Yes	Yes	Yes	No	Yes	Yes	Yes
Mugo (2014)* [71]	Yes	Yes	Yes	Yes	No	Yes	Yes	Yes
Kibengo (2013) [74]	Yes	Yes	Yes	Yes	No	Not Reported	Yes	Yes
Vardas (2010) [75]	Yes	Yes	Yes	Yes	No	Yes	Yes	Yes
Kibuuka (2009)* [76]	Yes	Yes	Yes	Yes	No	Not Reported	Not Reported	Yes
Peterson (2007) [78]	Yes	Yes	Yes	Yes	No	Yes	Yes	Yes
Feldblum (2008) [79]	Yes	Yes	Yes	Yes	No	No	Yes	Yes
Joachim (2016) [80]	Yes	Yes	Yes	Yes	No	Yes	Yes	Yes
Munseri (2015) [81]	Yes	Yes	Yes	Yes	No	Yes	Yes	Yes
Viegas (2018) [82]	Yes	Yes	Yes	Yes	No	No	Yes	Yes
Thigpen (2012)* [83]	Yes	Yes	Yes	Yes	No	Yes	Yes	Yes
Nel (2016a) [86]	Yes	Yes	Yes	Yes	No	Yes	Yes	Yes
Nel (2016b) [87]	Yes	Yes	Yes	Yes	No	Yes	Yes	Yes
Jaoko (2010) [88]	Yes	Yes	Yes	Yes	No	Yes	Yes	Yes
Schmidt (2014) [89]	Yes	Yes	Yes	Yes	No	Yes	Yes	Yes
